# Antibacterial properties and abrasion-stability: Development of a novel silver-compound material for orthodontic bracket application

**DOI:** 10.1007/s00056-022-00405-7

**Published:** 2022-07-18

**Authors:** Hannah Denis, Richard Werth, Andreas Greuling, Rainer Schwestka-Polly, Meike Stiesch, Viktoria Meyer-Kobbe, Katharina Doll

**Affiliations:** 1 Department of Dental Prosthetics and Biomedical Materials Science, Hannover Medical School, Carl-Neuberg-Str. 1, 30625 Hannover, Germany; 2 Lower Saxony Centre for Biomedical Engineering, Implant Research and Development (NIFE), Stadtfelddamm 34, 30625 Hannover, Germany; 3 Department of Orthodontics, Hannover Medical School, Carl-Neuberg-Str. 1, 30625 Hannover, Germany

**Keywords:** Silver infiltration, Biofilms, Abrasion resistance, Antibacterial orthodontic bracket material, Confocal laser scanning microscopy, Silber-Infiltration, Biofilm, Abrasionsstabil, Antibakterielles kieferorthopädisches Bracketmaterial, Konfokale Laser-Scanning-Mikroskopie

## Abstract

**Purpose:**

Bacteria-induced white spot lesions are a common side effect of modern orthodontic treatment. Therefore, there is a need for novel orthodontic bracket materials with antibacterial properties that also resist long-term abrasion. The aim of this study was to investigate the abrasion-stable antibacterial properties of a newly developed, thoroughly silver-infiltrated material for orthodontic bracket application in an in situ experiment.

**Methods:**

To generate the novel material, silver was vacuum-infiltrated into a sintered porous tungsten matrix. A tooth brushing simulation machine was used to perform abrasion equal to 2 years of tooth brushing. The material was characterized by energy dispersive X‑ray (EDX) analysis and roughness measurement. To test for antibacterial properties in situ, individual occlusal splints equipped with specimens were worn intraorally by 12 periodontal healthy patients for 48 h. After fluorescence staining, the quantitative biofilm volume and live/dead distribution of the initial biofilm formation were analyzed by confocal laser scanning microscopy (CLSM).

**Results:**

Silver was infiltrated homogeneously throughout the tungsten matrix. Toothbrush abrasion only slightly reduced the material’s thickness similar to conventional stainless steel bracket material and did not alter surface roughness. The new silver-modified material showed significantly reduced biofilm accumulation in situ. The effect was maintained even after abrasion.

**Conclusion:**

A promising, novel silver-infiltrated abrasion-stable material for use as orthodontic brackets, which also exhibit strong antibacterial properties on in situ grown oral biofilms, was developed. The strong antibacterial properties were maintained even after surface abrasion simulated with long-term toothbrushing.

## Abstract (de)

**Zielsetzung:**

Bakterieninduzierte „white spot lesions“ sind eine der häufigsten Komplikationen moderner kieferorthopädischer Behandlungen. Daher besteht großes Interesse an neuen kieferorthopädischen Bracketmaterialien mit antibakteriellen Eigenschaften, die gleichzeitig abrasionsstabil sind. Ziel dieser In-situ-Studie war die Untersuchung der antibakteriellen Eigenschaften in Abhängigkeit zur Abrasion eines neu entwickelten, vollständig silberinfiltrierten Materials für die kieferorthopädische Anwendung.

**Methoden:**

Zur Herstellung des neuartigen Materials wurde Silber mittels Vakuuminfiltration in eine gesinterte, poröse Wolfram-Matrix eingebracht. Um die Abrasion durch Zähneputzen über einen Zeitraum von 2 Jahren nachzustellen, wurde eine Zahnputzsimulationsmaschine genutzt. Das Material wurde im Anschluss mittels energiedispersiver Röntgenspektroskopie (EDX) und Rauheitsmessung analysiert. Um die antibakteriellen Eigenschaften in situ zu untersuchen, wurden die Probekörper an individuellen Aufbissschienen befestigt und von 12 parodontal gesunden Probanden für 48 h intraoral getragen. Nach anschließender Fluoreszenzfärbung wurden das Biofilmvolumen sowie die Lebend-tot-Verteilung durch konfokale Laserscanningmikroskopie (CLSM) quantifiziert.

**Ergebnisse:**

Das Silber konnte homogen in die Wolfram-Matrix infiltriert werden. Das Material widerstand der Zahnputz-Abrasion in vergleichbarem Maße wie konventionelles Edelstahl-Bracketmaterial und auch die Oberflächenrauheit veränderte sich nicht. Eine signifikante Reduktion der Biofilmakkumulation konnte auf dem neuen silbermodifizierten Material nachgewiesen werden. Dieser Effekt blieb auch nach der Abrasion erhalten.

**Schlussfolgerung:**

Es konnte ein neues silberinfiltriertes, abrasionsstabiles Material mit vielversprechenden Eigenschaften für die Anwendung als kieferorthopädisches Bracketmaterial entwickelt werden, das starke antibakterielle Eigenschaften gegenüber oralen Biofilmen in situ aufweist. Dieser antibakterielle Effekt bleibt auch nach Langzeit-Abrasion mit einer Zahnputz-Simulationsmaschine erhalten.

## Introduction

Dental plaque, which can be found on tooth surfaces in the oral cavity, is a three-dimensional, matrix-enclosed structure, comprising up to 70% bacteria of several hundred different species that interact with each other [23, 35, 44]. If this biofilm is not continuously removed by daily oral care, it promotes the development of severe oral diseases such as caries or periodontitis [14]. Bacteria in dental plaque take up carbohydrates from food and metabolize them to inorganic acids. These acids irreversibly dissolve components from the dental enamel, such as calcium and phosphate, resulting in a roughened tooth surface. The process additionally causes macroscopically visible decalcifications underneath the plaque, called white spot lesions (WSL). WSL are considered active initial caries lesions, which can progress to caries, the most common oral disease [4, 6, 61].

In Germany, up to 45% of children and 58% of young adults receive orthodontic treatment [12]. Multibracket appliances impede daily oral care and reduce the self-cleaning effect in the oral cavity, thus, providing ideal retention surfaces for bacteria [15, 20, 35, 45]. Roughness and the kind of material of the orthodontic brackets can also have an influence on bacterial colonization [50]. The prevalence of caries in 15 year olds was significantly greater compared to 12 year olds due to the eruption of permanent teeth and the closing of the dentition [12]. Furthermore, there is a correlation between the onset of puberty and neglecting oral hygiene [12]. Dental plaque accumulation frequently results in circular WSL around brackets, which significantly increase the frequency of caries [7, 60, 65, 68]. The incidence of WSL and cavitated lesions correlates with the duration of orthodontic treatment and the personal oral hygiene standards. Despite preventive oral hygiene, WSL in orthodontic treatments are still prevalent in up to 72.9% of patients [25, 57]. In addition, biofilm accumulation on orthodontic brackets increases the probability of supragingival plaque, which may lead to gingivitis or, in the worst case, even periodontitis with loss of surrounding bone structure [18, 49, 56]. These data underline the clinical necessity for effective dental plaque prevention in orthodontic treatment [25, 27, 57].

A potential approach for preventing bacterial biofilm accumulation could be the specific modification of the bracket surface—the initial attachment site for bacteria [17]. In this context, using silver has emerged as an interesting material modification as it shows strong intrinsic antibacterial properties and is already frequently used in various areas of dentistry [8, 26, 46–48, 59, 69, 75]. Silver ions interfere with bacterial metabolism by impairing DNA replication, reducing certain protein activities and inhibiting important enzymes of the respiratory chain [28, 53]. In implantology, the antibacterial effect of silver is already applied in coatings composed of porous silica and silver nanoparticles [46], or silver nanocoatings on implants. Thereby, the bacterial growth in the surrounding tissue can be successfully inhibited, preventing peri-implantitis [8]. In recent years, orthodontic research also investigated materials with silver-based antibacterial effects [22]. Several studies focusing on coatings of conventional stainless steel bracket material with silver compounds have been conducted. The application of a silver–platinum electroplate coating exerted a strong antibacterial effect against the colonization with *Streptococcus mutans* and *Aggregatibacter actinomycetemcomitans *[59]. Antiadhesive and antibacterial properties were further demonstrated against *S. mutans* and *Porphyromonas gingivalis* on titanium oxide silver coatings [26]. This characteristic was also detected for nano silver–titanium dioxide surfaces [75]. In addition, silver nanocoatings were shown to prevent WSL independently of patient cooperation in an in silico study over 75 days [47].

However, all silver-modifications examined in these previous studies were surface material coatings. Orthodontic brackets are exposed to severe mechanical friction by daily tooth brushing and it was shown that pure material coatings, e.g., polytetrafluoroethylene (PTFE), are not able to withstand this abrasion [19, 31]. Since similar results can be expected for silver coatings, this issue has already been addressed by the authors in a previous study by means of silver implantation via plasma immersion ion implantation and deposition (PIIID) [48]. Although this study demonstrated bactericidal properties in addition to antibacterial effects, the calculated penetration depth of silver ions was only up to 9 nm, which is most likely too low to withstand long-term abrasion [48, 69]. Therefore, a potential abrasion-stable antibacterial material bracket material has not yet been developed.

To overcome these limitations a material that is comparably hard and stable, but also completely silver-penetrated is required. Such material would resist most abrasion but at the same time expose silver on its surface even if slightly abraded. A possibility for creating this material could be using a stable metallic framework, e.g., generated by sintering, with subsequent silver infiltration. As tungsten sintered pellets were available, this material was used for the investigation. The sintered metal exhibits high tensile strength and density [62, 76]. It has already been used as coils for transcatheter embolization of pathological blood vessels and for microwire neural electrodes implanted in animals. Furthermore, tungsten is frequently used in alloys for cardiovascular stents, orthopedic joint replacements and dental prosthetics [62, 74].

In this study, a promising, first-of-its-kind material for orthodontic brackets, which exploits a sintered porous tungsten matrix completely infiltrated with silver to ensure long-term stable antibacterial effects, was investigated. In order to analyze long-term stability, the novel material was exposed to mechanical friction and degradation using a toothbrush simulation machine. Before and after abrasion, the novel material was characterized using energy dispersive X‑ray (EDX) analysis as well as roughness and wall thickness quantification. The antibacterial effect was examined in situ by exposing the material on an occlusal splint intraorally for 48 h in 12 subjects. Biofilm volume and live/dead ratio was quantified using confocal laser-scanning microscopy (CLSM).

## Materials and methods

### Fabrication and characterization of silver-infiltrated specimens

The newly developed material is a novel type of silver-modified metal. Tungsten was sintered to porous round specimens of 19.3 mm diameter and 2 mm thickness. In a vacuum-melting process, the specimens were subsequently infiltrated with silver. The ratio of tungsten to silver was set at 85/15% or 80/20% for later division into two groups for analysis. Using a belt grinding machine, the specimens were ground to the desired nominal thickness of 0.64 mm. The specimens were laser cut to the desired nominal size of 5 mm × 5 mm. For controls, commercially available orthodontic bracket stainless steel (Forestadent Bernhard Förster, Pforzheim, Germany) was laser-cut into specimens of the same size. The material composition of each specimen was analyzed with an EDX scanning electron microscope (Tescan Vega 3 with EDX, Brno, Czech Republic). The roughness of the silver-infiltrated material and the unmodified stainless steel bracket material was examined using a CLSM (Keyence VK-X100 series, Keyence Deutschland, Neu-Isenburg, Germany). The wall thickness was measured with a digital micrometer.

### Tooth brushing simulation machine

**Fig. 1:**
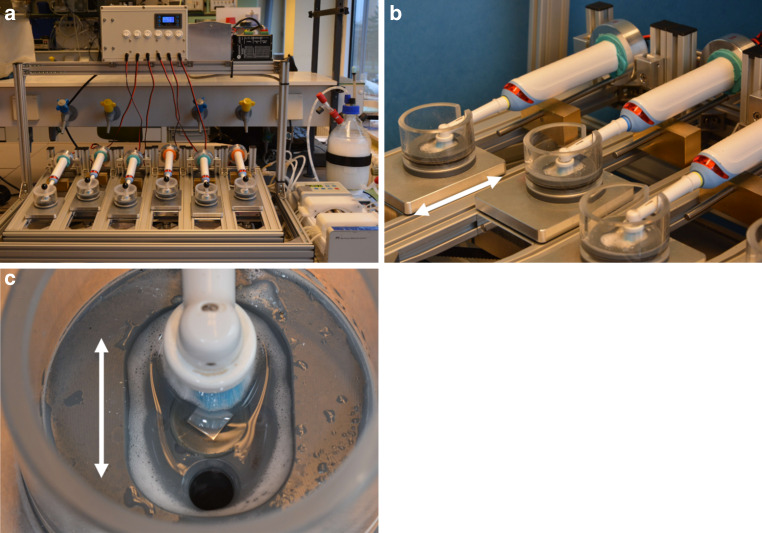
**a** Custom-built automated tooth brushing simulation machine. **b** Forward and backward movement of the toothbrushes (*white arrow*). **c** Close-up of the box moving back and forth (*white arrow*); the toothbrush heads performed an additional rotating oscillating motion on the test specimen in its holder **a** Selbstkonstruierte automatische Zahnputzsimulationsmaschine. **b** Vor- und Zurück-Bewegung der Zahnbürsten (*weißer Pfeil*). **c** Nahaufnahme der sich vor- und zurückbewegenden Probenkammer (*weißer Pfeil*); der Zahnbürstenkopf führt zusätzlich rotierend-oszillierende Bewegungen auf der Probe durch

The tooth brushing simulation machine used in this study was self-built and designed to imitate the daily brushing of teeth (Fig. 1a). It consisted of six electric toothbrushes (Professional Care Pro 1000, Oral‑B, Procter & Gamble, Schwalbach am Taunus, Germany), in which the battery was replaced with a grid-based power supply. The electric brushes were mounted on a metal frame, which moves forward and backward at 0.5 Hz over a range of 10 mm (Fig. 1b). The brushing heads rest in a small box performing a rotating–oscillating movement. The contact pressure exerted on the specimen could be adjusted with a weight and was individually set to 2 N, which is within the recommended application range [9, 63]. Each box had two holes, which allowed for a saliva-like solution to be pumped in and out via a peristaltic pump (IPC Ismatec, Cole-Parmer, Wertheim, Germany). As the samples needed to be disc-shaped to be fixed in a notch between the two pumping holes, a costume-built specimen holder made from polyether ether ketone was used (Fig. 1c). When operating, the cases moved forward and backward, which also distributed fresh saliva-like solution on the sample surface.

The integrated tube system was filled with a solution composed of water, 250 mg/L mucin (Sigma Aldrich Chemie, München, Germany) and 10 g/L toothpaste (Oral-B®, Gum& Enamel Pro-Repair Original Zahnpasta, Procter & Gamble, Schwalbach, Germany) to imitate human saliva while tooth brushing. The solution was circularly pumped with a velocity of 3 ml/min to meet average salivary flow based on previous research [24, 38]. Specimens were treated for 2 h to simulate tooth brushing of 2 years based on an average treatment of a single tooth side for 2 s when tooth brushing twice a day for 2 min [33, 63]. Afterwards, the test specimens were rinsed with distilled water before being used for further experiments.

### Test subject selection

The Hannover Medical School approved the ethic vote for the envisaged study (ethic vote no. 8570_BO_S2019 “Development and antibacterial characterization of a novel, silver-modified and abrasion-resistant bracket material”). Involved in the study were 12 healthy patients aged 21–30 years, including 6 female and 6 male participants. The prerequisite for participation in the study was a healthy periodontal condition, which was initially examined by periodontal screening. The screening process included the modified approximal plaque index (API), the modified sulcus bleeding index (SBI) and the probing depths (PD) [34]. Exclusion criteria were pregnancy, general diseases, smoking, removable dentures and antibiotics taken less than 6 weeks prior to the start of the study. The subjects were informed in detail about the study by a face-to-face explanation and a supplemental information sheet. The patient’s permission was confirmed by signature on a consent form. The results were anonymized and randomized.

### Splint design and examination period

**Fig. 2:**
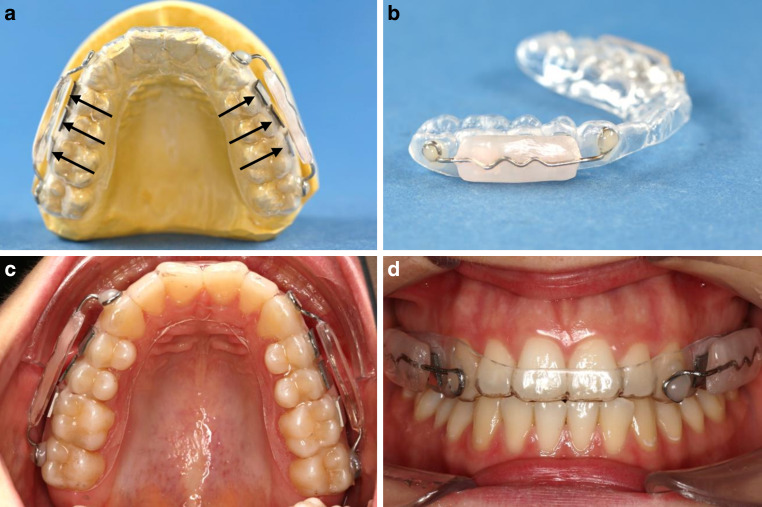
**a** Occlusal splint with laterally fixed specimens (*arrows*) and vestibular shields on a plaster model. **b** Side view on a manufactured buccal-attached plastic shield. **c** Occlusal splint inserted intraorally. **d** Coronal front view of an integrated splint Design der Aufbissschienen. **a** Aufbissschiene mit lateral befestigten Probekörpern (*Pfeile*) und vestibulären Schilden auf einem Gipsmodell. **b** Seitansicht eines bukkalen Plastikschilds. **c** Oral eingesetzte Aufbissschiene. **d** Koronale Frontansicht der eingesetzten Schiene

The design is based on the occlusal splints used in the study of Meyer-Kobbe et al. [48]. Impressions of the upper jaw were taken with alginate (Alginoplast®, Kulzer, Hanau, Germany) and poured with class 3 plaster (Sheraalpin, Shera Werkstoff-Technologie, Lemförde, Germany). Using the plaster model and thermoplastic deep drawing procedure (Erkodur, Erkodent® Erich Koop, Pfalzgrafenweiler, Germany), an occlusal splint of plastic was prepared individually (Fig. 2). To retract the tongue, lips and cheeks from the test specimens, additional shield-like plastic plates were manufactured exerting the sprinkle and spray technology with clear plastic (Orthocryl®, Dentaurum, Ispringen, Germany) [42]. To ensure reliable stability for the plastic plates, a support with a corresponding wire foil of a thickness of 0.9 cm (remanium® wire, Dentaurum, Ispringen, Germany) was bent, terminated with a triangle bending. This construction guaranteed a mere millimeter-wide gap between the occlusal splint and the shields for a constant salvation over the samples (Fig. 2a, b). On both sides at the position of the posterior teeth, the individual occlusal splint was roughened with sandpaper (Henry Schein®, Langen, Germany). Light cure adhesive paste (Transbond™ XT, 3M Unitek, Neuss, Germany) and light cure adhesive primer (Transbond™ XT, 3M Unitek, Neuss, Germany) were used to attach the plastic shields. Both groups of silver-infiltrated specimens and the control groups, each before and after abrasion, were randomly attached per test subject. The specimens were placed with flowable composite (Tetric EvoFlow A3, Ivoclar Vivadent, Schaan, Lichtenstein) on the occlusal splints in the first and second quadrants in the premolar and molar region (Fig. 2c, d).

The splint equipped with the specimens was worn for 48 h by the participants of the study. During this time, oral hygiene was completely suspended and alcohol consumption was prohibited. For eating, the splint was removed for up to 40 min, being stored in a humid environment of a wet cloth. After 48 h, the shields were clipped off and the samples were carefully removed with tweezers for subsequent analysis.

### Microscopic analysis of biofilm formation

The biofilm covered specimens were first transferred to phosphate-buffered saline (Biochrom, Berlin, Germany) to remove nonbound bacteria. To assess biofilm formation, the dental plaque was labelled with the Live/Dead Staining® BacLight™ Bacterial Viability Kit (Thermo Fisher Scientific, Braunschweig, Germany), consisting of the two fluorescent dyes Syto9 and propidium iodide, according to the manufacturer’s instructions. Afterwards, they were fixed with 2.5% glutardialdehyde (Carl Roth, Karlsruhe, Germany). The specimens were stored in phosphate-buffered saline for microscopy. Using a confocal laser scanning microscope (TCS SP8, Leica Microsystems, Wetzlar, Germany), three-dimensional images of the biofilms were taken at a magnification of 40 × with a z-step-size of 3 µm at five defined positions of the specimen. Syto9 was excited using a 488 nm laser line and emission was detected at 500–550 nm. Propidium iodide was excited using a 552 nm laser line and emission was detected at 600–700 nm. Applying the Imaris software package (Imaris 8.4, Bitplane, Zurich, Switzerland), the resulting images were analyzed for biofilm volume and live/dead distribution. Syto9 signal was considered “live” and propidium iodide signal was considered “dead”. Colocalized staining was also considered “dead”, as propidium iodide was able to penetrate the bacteria, and subtracted from the amount of “live” biofilm.

### Statistical analysis

The GraphPad Prism software 8.4 (GraphPad Software, Inc., La Jolla, CA, USA) was used for statistical analysis and data visualization. Significant differences between the different materials before and after abrasion were assessed by repeated-measures two-way analysis of variance (ANOVA) with Bonferroni’s correction for multiple comparisons. To compare wall thickness reduction (difference before and after abrasion) between the different materials, Kruskal–Wallis test with Dunn’s correction for multiple comparison was performed. Significance level was set to α = 0.05.

## Results

### Characterization of silver-infiltrated test specimens

**Fig. 3:**
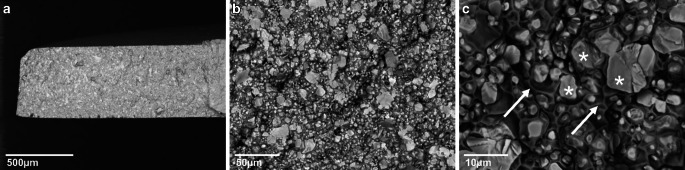
Scanning electron microscopy images of cross-sections of the new material at different magnifications. Scale bars corresponding to **a** 500 µm, **b** 50 µm and **c** 10 µm, respectively. The tungsten matrix is indicated by *stars* and silver by *arrows* Rasterelektronenmikroskopische Bilder von Schnittflächen des neuen Materials in unterschiedlichen Vergrößerungen. Die Maßstabsbalken entsprechen **a** 500 µm, **b** 50 µm und **c** 10 µm. Die Wolfram-Matrix ist durch *Sternchen*, das infiltrierte Silber durch *Pfeile* gekennzeichnet

First, EDX analysis was conducted in order to characterize the newly produced silver-infiltrated and the control material. The unmodified control material consisted on average of 68.6% iron (Fe), 17.72% chromium (Cr) and 13.66% manganese (Mn). This composition is common for nickel-free fixed appliances with the material number 1.4456 [71]. Regarding the novel test specimens, EDX analysis confirmed a combination of tungsten and silver (Ag), and yielded silver contents ranging from 7.24 to 14.59%. For further analysis, the specimens were divided into two groups: low Ag (7.24–9.72%) and high Ag (9.76–14.59%). Samples of both groups were paired correspondingly, so that the difference of silver content was as high as possible. In order to investigate the distribution of the silver within the new material, a cross section was conducted. Scanning electron microscopy images showed a net-like structure of the tungsten matrix (Fig. 3a–c, stars). It is continuously surrounded by the infiltrated silver (arrows).

### Abrasion test on the specimens

**Fig. 4:**
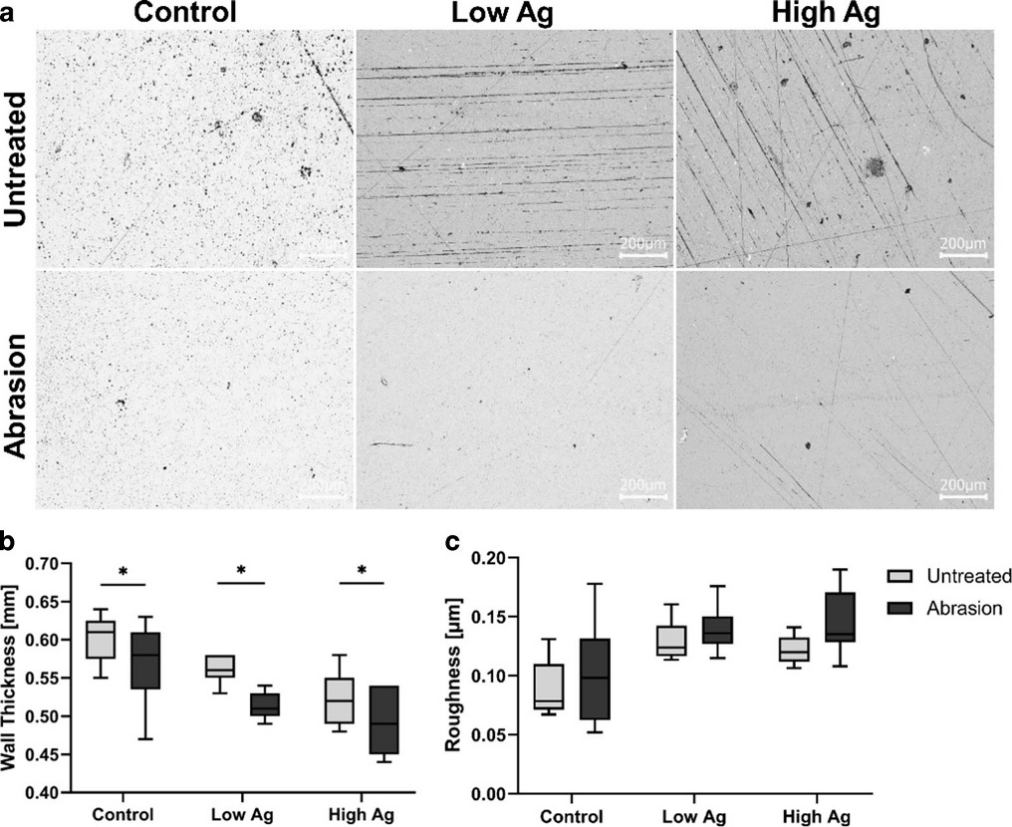
Abrasion-dependent material characterization. **a** Microscopic image of the material’s surfaces. **b** Wall thickness, and **c** roughness of indicated specimens before (untreated) and after abrasion. *Asterisk* statistically significant differences at *p* ≤ 0.05 Materialcharakterisierung in Abhängigkeit zur Abrasion. **a** Mikroskopische Aufnahmen der Materialoberfläche. **b** Wandstärke und **c** Rauheit der unterschiedlichen Probekörper vor und nach der Abrasion. *Asterisk* statistisch signifikante Unterschiede mit *p* ≤ 0,05

After treating the novel silver-infiltrated and control specimens in the tooth brushing simulation machine, material wall thickness and roughness were examined. In addition, exemplary microscopic images were taken with 100 × magnification (Fig. 4a). The wall thickness of control samples was 0.6 ± 0.03 mm before abrasion and 0.57 ± 0.05 mm afterwards. Specimens of the low Ag group were 0.56 ± 0.02 mm and 0.51 ± 0.02 mm thick, respectively. The high Ag samples had a wall thickness of 0.52 ± 0.03 mm before and 0.49 ± 0.04 mm after abrasion. Comparing the untreated with the abraded specimens, the sample thickness decreased only slightly, even though the difference was statistically significant, within all groups upon treatment with the tooth brushing simulation machine (Fig. 4b). This wall thickness reduction was statistically equivalent between the groups (*p* = 0.34–0.99).

The average surface roughness (Ra) was determined by CLSM. For controls, Ra was 0.089 ± 0.024 µm before and 0.098 ± 0.042 µm after abrasion. The Ra values were 0.145 ± 0.016 µm and 0.139 ± 0.018 µm for low Ag, and 0.123 ± 0.012 µm and 0.145 ± 0.026 µm for high Ag specimens before and after abrasion treatment, respectively. There were no significant differences in roughness before and after experimental abrasion within the respective groups (Fig. 4c).

### Reduced biofilm formation on silver-infiltrated test specimens in situ

In this study, only healthy young adults with conscientious regular oral hygiene participated. The test subjects were aged between 21 and 30 years (23.9 ± 2.4 years), weighed 50 to 95 kg (71.9 ± 15.2 kg) and their height ranged from 146 to 195 cm (178.5 ± 12.3 cm). The periodontal screening resulted in an API of 10.0 ± 4.5%, a SBI of 10.8 ± 7.1% and a PD of 1.4 ± 0.1 mm.

**Fig. 5:**
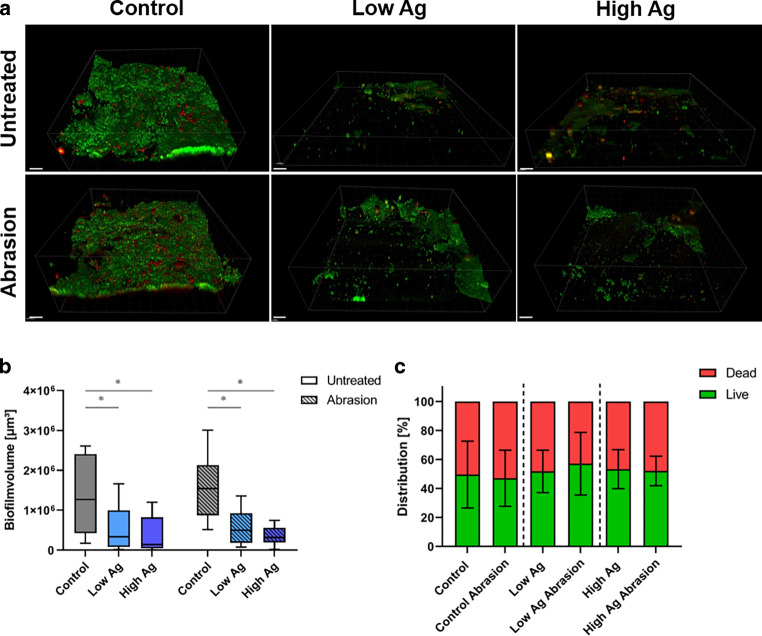
Quantification of biofilm formation on occlusal splints. **a** Three-dimensional confocal laser-scanning microscopy (CLSM) image reconstruction of in situ grown biofilms on different materials. Living bacteria stained *green* and dead bacteria stained *red/orange*. Scale bars: 30 µm.** b** Box plot diagram of biofilm volume and **c** mean ± standard deviation of bacterial live/dead distribution on the different materials before (untreated) and after abrasion. *Asterisk* statistically significant differences at *p* ≤ 0.05 Quantifizierung der Biofilmbildung auf den Aufbissschienen. **a** Dreidimensionale confocal laser-scanning microscopy (CLSM)-Bild-Rekonstruktion von in situ gebildeten Biofilmen auf den unterschiedlichen Materialien. Lebende Bakterien sind *grün*, tote Bakterien *rot/orange* angefärbt. Maßstabsbalken: 30 µm. **b** Box-Plot-Diagramm des Biofilmvolumens und **c** Mittelwert ± Standardabweichung der bakteriellen Lebend/Tot-Verteilung auf den unterschiedlichen Materialien vor und nach der Abrasion. *Asterisk* statistisch signifikante Unterschiede mit *p* ≤ 0,05

After wearing the splints for 48 h, biofilm formation was immediately assessed by CLSM. The in situ grown biofilm consisted of bacteria and mucosal cells (Fig. 5a). The biofilm volume and live/dead distribution were quantified (Fig. 5b, c). The control samples had a biofilm volume per image of 1.3 × 10^6^ ± 9.7 × 10^5^ µm^3^ before abrasion and 1.6 × 10^6^ ± 8.4 × 10^5^ µm^3^ afterwards. In comparison, the biofilm was significantly reduced in the low Ag group. The specimens showed biofilm amounts per image of initially 5.3 × 10^5^ ± 5.4 × 10^5^ µm^3^ and 6.0 × 10^5^ ± 4.5 × 10^5^ µm^3^ after treatment with the toothbrush simulator. Likewise, a significantly reduced biofilm volume could be detected in the high Ag group compared to the respective controls. Initially, the untreated group had a biofilm volume of 4.2 × 10^5^ ± 4.5 × 10^5^ µm^3^, whereas it was 3.6 × 10^5^ ± 2.3 × 10^5^ µm^3^ after abrasion. The biofilm was reduced on average by 60.8% before and 63.6% after abrasion in the low Ag group and 68.6% before and 78.1% after abrasion in the high Ag group. Thus, low Ag and high Ag specimens significantly reduced biofilm volume, both before and after abrasion.

In addition, quantification of the live/dead distribution was performed to investigate a potential bactericidal effect of the novel silver-modified material (Fig. 5c). Before abrasion, in the control group a distribution of 49.6% living and 50.4% dead bacteria was determined. The distribution was 51.8/48.2% in the low Ag group and 53.3/46.7% in the high Ag group for live/dead bacteria, respectively. After abrasion the live/dead distribution was almost similar. No statistically significant differences between any of the investigated groups could be detected.

## Discussion

White spot lesions are one of the most common complications of modern orthodontic treatment [4, 6, 61]. They are caused by bacterial accumulation and acid production around the brackets [15, 20, 35, 45]. The aim of this study was to investigate the antibacterial effect of a novel silver-infiltrated material for orthodontic brackets. In contrast to previous studies, a specific focus was additionally laid on its abrasion stability.

For fabrication, silver was vacuum-infiltrated into a sintered porous tungsten matrix. To the best of our knowledge, this is the first study to describe the combination of sintering and vacuum-infiltration for the generation of silver-modified materials. The success of the manufacturing process was examined by EDX scanning electron microscopy analysis revealing that the sintering process yielded a tungsten matrix completely interspersed with pores. In these pores of different volumes, silver was infiltrated under vacuum-melting. The results did not yield the expected silver content of 15 und 20%, but were comparably lower. If higher silver proportions would be necessary in the compound material, the infiltration process would need to be adjusted in further experiments. However, even this lower concentrated silver was distributed in all three dimensions throughout the matrix as confirmed by cross-section analyses. This differs from previous silver-modified bracket materials coated or penetrated only to a low depth. In prior modifications, such as silver-platinum electroplate coatings, titanium oxide silver coatings, nano silver–titanium dioxide surfaces, silver nanocoatings and PTFE coatings, silver was only located on the surface [26, 31, 47, 59, 75]. In a PIIID-modified bracket material, the calculated penetration depth of silver ions was also only up to 9 nm [48]. The new material developed in this study provided a clearly improved silver distribution within the material and, thus, fulfilled the prerequisites for an abrasion-stable antibacterial bracket material. Tungsten is already applied in neuroscience, vascular medicine, radiology, orthopedics and prosthetics due to high density, radiopacity, tensile strength and yield strength. When implanted, it has no long-term resistance to corrosion, which is accelerated by the immune system [62, 74]. The mechanisms of the corrosion process have not yet been thoroughly researched [62, 76]. However, in studies with nanoparticle coatings of tungsten disulfide on orthodontic wires, low attrition and reduced friction have been shown [32, 55]. In addition, cytotoxicity analysis of tungsten nanoparticles indicated biocompatibility. For the use as bracket material, the use of tungsten is considered innocuous, as the new material is not implanted but only bonded on the surface of the teeth for a limited period of time [52].

To test the abrasion stability of the new material, a self-constructed tooth brushing simulation machine was used. The set-up simulates realistic tooth brushing movements and flow of saliva and toothpaste. The brushing time was calculated to resemble 2 years of tooth brushing, since treatment with fixed orthodontic appliances usually takes about 1.5–2 years [52]. Thus, the experimental abrasion in this study reflects the natural exposure of bracket materials. The saliva-like mixture was produced from mucin, a glycosylated protein that is a major component of human saliva [38]. In contrast to collecting native saliva from volunteers, this allows for more reproducible experimental conditions and a larger liquid volume. The set-up furthermore applies the recommendations for daily oral care, to be specific, using an electric toothbrush and toothpaste. Electric toothbrushes with an oscillating-rotating motion significantly reduce the incidence of gingivitis compared to manual toothbrushes [33]. Fluoride toothpastes are recommended for their acceptability and for caries prevention [33]. By means of a micrometer, the material thickness was quantified before and after abrasion. The toothbrush simulation machine reduced the wall thickness of the specimens by 30–50 µm only. This reduction was equal between the newly developed material and conventional stainless steel bracket material. As the latter is known for its high abrasion-stability, the new material confirmed to be abrasion-stable as well.

In order to create equal baseline conditions for biofilm analyses, the roughness of each specimen was analyzed. Material roughness has a major influence on bacterial adhesion with increasing values promoting bacterial colonization due to more available surface [64]. There were no significant differences in roughness within and between the groups. The roughness of the materials did not change due to toothbrushing, verifying their abrasion-stability. In addition, a major influence of roughness of either specimen on bacterial adhesion can be excluded. Therefore, in the following in situ examination, the antibacterial effect should be primarily related to the different material compositions.

The antibacterial properties of the novel silver-modified material were tested in situ by participants wearing occlusal splints equipped with the test specimens. To confirm healthy oral conditions, the participants underwent a periodontal screening prior to this study, whereby SBI did not exceed 10%, the PD did not extend beyond 3 mm and the API was less than 25% [14, 70, 72]. Thus, based on the data collected, healthy oral condition of the test subjects could be verified. In addition, further risk factors were excluded that could alter the composition of the microbial flora, such as general diseases, antibiotic use, pregnancy, alcohol consumption and smoking [2, 10, 12, 16, 30, 39, 54, 73]. The splint design has already been used in previous studies of fix specimens [5, 21, 31, 43, 48]. The test specimens are not fixed directly to the enamel, which could lead to enamel cracks when being removed [1, 41]. In this experimental set-up, the splint design of Meyer-Kobbe et al. [48] was used, including laterally attached shield-like plastic plates to prevent the cheek and tongue from contacting the specimens. This prevents the biofilm formation on the specimens from being affected by shear forces resulting in reduced biofilm accumulation. In addition, saliva can circulate unimpededly through the gap between the plastic plates and the specimens [48]. Precluding the possibility of position-dependent biofilm coverage [3, 5], the specimens were fixed in a random order on the individual occlusal splints. Each material was attached to each position on the splint at least once. Thus, position-specific effects could be excluded. The wearing time was set to 48 h, since formation of individual initial biofilm usually occurs within this period of time and this wearing time has already been successfully used in previous studies [3, 5, 43, 48].

For the analysis of the biofilm, an established CLSM method for the quantification of initial biofilm formation was employed. This allows a nearly native detection of biofilm morphology and simultaneous examination of live/dead distribution [22, 29, 51, 66]. In addition to bacterial cells, human cells, presumably gingival epithelial cells, appeared on the surfaces of the stained biofilms. Human gingival epithelial cells can be colonized by oral bacteria and be integrated into the biofilm [67]. This phenomenon has already been observed in other studies, where the human cells were likewise regarded as components of the biofilm [48].

The quantification of biofilms revealed a significant reduction in plaque accumulation in terms of biofilm volume on all silver-infiltrated material surfaces compared to conventional stainless steel bracket material. As an influence of the material’s roughness was excluded, this effect can be attributed to the silver content of the novel material. Interaction between silver and components of the bacterial membrane leads to membrane damage and disruption of intracellular metabolic activity. Protein activity is reduced and bacterial DNA loses its ability to replicate. Consequently, important enzymes of the respiratory chain are inhibited and cell death ensues [28, 40, 53]. The biofilm-reducing effect has already been described using silver coatings such as silver–platinum electroplate coating, titanium oxide–silver coatings, nano silver–titanium dioxide surfaces and silver nanocoatings [26, 47, 59, 75]. Besides their strong antibacterial effect, it should be taken into account that using nano silver-coated orthodontic brackets in an animal model resulted in elevated nano silver levels in saliva and serum [47]. Nano silver is also claimed to induce silver resistance in pathogenic germs as it is used more and more frequently in consumer goods [13]. The exact mechanism as well as the cytotoxic concentration are still under investigation and seem to depend on the specific size of the applied silver [13]. Thus, it might be that ionic silver, which should be responsible for the antibacterial effect observed in this study, is less toxic than nano silver coatings. However, before applying the material developed in this study for orthodontic treatment, toxicity analysis for this specific setup is inevitable.

In the present study, the biofilm volume was reduced by 60% to almost 80% on the newly developed material. This is lower than in a recent study, where a PIIID silver-modified surface, an electroplated silver layer and a physical vapor deposition (PVD) silver coating led to a biofilm reduction of up to 95% [48]. Most probably, this is due to the different types of material modification: Whereas the recent study led to almost continuous silver layers on the surfaces, the material analyzed here was a compound of silver and tungsten. Interestingly, the low Ag and high Ag group of the novel material did not differ in the antibacterial effect. It has already been shown that even low concentrations of silver (5%) are sufficient for effective biofilm prevention [36]. Therefore, for the silver concentrations used in this study (7–15%) the saturation level may have been reached with no further effect with increasing content. With regard for a prospective clinical application as bracket material, this would require lower amounts of silver for the fabrication process. Future studies could evaluate whether even lower concentrations may maintain the same antibacterial effect. In addition, the silver concentration in the novel material will be distinctly lower than in an already accredited product for tooth conservation with silver content of approximately 50% (Ketac silver Aplicap, 3M Deutschland, Neuss, Germany). These are promising preconditions for a potential medical device approval of the novel material [58].

Importantly, there was also no difference when comparing the initial biofilm formation on untreated and abraded specimens. In both cases, the biofilm volume was significantly lower compared to the control material. Thus, the antibacterial properties of the novel material were maintained after simulating 2 years of mechanical tooth brushing abrasion. Most probably, this is due to the abrasion-stability and successful continuous silver-infiltration throughout the material [27, 52]. Dental surfaces are exposed to high shear forces in the mouth. Surface coatings used in previous studies, such as PTFE coatings, have been demonstrated to become partially abraded and rubbed off upon exposure [19]. Long-term stability could, thus, not be guaranteed. To the best of our knowledge, the antibacterial effect of the new material of this study is the first that demonstrated long-term stability over the period of a conventional orthodontic treatment.

To analyze for an additional antibacterial effect of the novel material, as detected for the previously analyzed PIIID silver-modified surfaces [48], the biofilm live/dead ratio was determined. For this purpose, a staining with two fluorescent dyes with different membrane permeability was applied. All bacteria are stained green, but only those with impaired membrane—defined as dead—take up the red dye, which ideally replaces the green dye [11, 37]. An almost unchanged 50/50 distribution of living and dead cells was detected in all groups. Accordingly, the novel material does not exhibit an additional bactericidal effect. These results may be biased by the human cells. The mucosal cells on the specimens, due to possessing different membrane properties than bacteria, also absorb the red dye of the live/dead staining even when they are still alive. As they are several folds greater than bacterial cells, they will account for the major proportion of red staining. Therefore, only a major bactericidal effect would have been detectable. In fact, in the previous study by Meyer-Kobbe et al. [48] the bactericidal effect was detected in a similar experimental set-up. To examine the properties of the novel silver-infiltrated material in more detail, future studies should apply further methods to analyze the bacterial composition and molecular pathways of the antibacterial effect.

## Conclusion

In this study, a quantitative analysis of the initial biofilm formation on a novel silver-infiltrated material was successfully performed under in situ conditions. The material exploits for the first time the combination of sintering and silver vacuum-infiltration to ensure a long-term antibacterial effect as required for bracket materials in orthodontics. A significant antibacterial effect was demonstrated, which was maintained after prolonged abrasion using a tooth brushing simulation machine due to its high stability. In further studies, consideration must be given to the clinical implementation of this novel material for orthodontic brackets regarding production and specific application. The fabrication for this study was a pilot process and will require more research on economic feasibility and up-scaling for translation to orthodontic application. Furthermore, it needs to be considered whether the current framework metal tungsten should be substituted in favor of the established orthodontic stainless steel. Nevertheless, the novel material of this study exhibits most promising characteristics to significantly reduce intraoral biofilm formation on orthodontic brackets. It could contribute to fewer WSL in patients receiving orthodontic treatment and reduce the risk of subsequent diseases, such as caries and periodontitis.
